# The lipid peroxidation-derived DNA adduct γ-OHPdG as a diagnostic and prognostic biomarker in hepatocellular carcinoma

**DOI:** 10.18632/aging.204910

**Published:** 2023-07-28

**Authors:** Qiwei Cao, Yazhou Zhang, Hongtao Liu, Yuxia Cheng, Mingxin Liu, Hai Zhao, Ruixue Tang, Junying Sun, Sophia Xu, Bing Sun, Qing Sun

**Affiliations:** 1 Department of Pathology, The First Affiliated Hospital of Shandong First Medical University and Shandong Provincial Qianfoshan Hospital, Shandong Medicine and Health Key Laboratory of Clinical Pathology, Shandong Lung Cancer Institute, Shandong Institute of Nephrology, Jinan, Shandong Province, P.R. China; 2 Department of Pathology, The Affiliated Provincial Hospital of Shandong First Medical University, Jinan, Shandong Province, P.R. China; 3 Shandong Life Science and Technology Ltd., Dezhou, Shandong Province, P.R. China; 4 University of California San Diego, San Diego, CA 92093, USA; 5 Lombardi Comprehensive Cancer Center, Georgetown University Medical Center, Washington DC 20007, USA

**Keywords:** hepatocellular carcinoma, cirrhosis, γ-OHPdG, occurrence, prognosis

## Abstract

Purpose: Chronic inflammation and lipid peroxidation (LPO) are associated with the pathogenesis of hepatocellular carcinoma (HCC), and γ-hydroxy-1, N^2^-propanodeoxyguanosine (γ-OHPdG) is a promutagenic DNA adduct derived from LPO. This study aimed to examine the relationship between γ-OHPdG and the progression of liver carcinogenesis.

Methods: Primary HCC specimens were obtained from 228 patients and cirrhosis specimens from 46 patients. The patients were followed up with after surgery via outpatient visits and telephone calls. The levels of γ-OHPdG were determined by immunohistochemical analysis in the carcinomatous tissues together with adjacent and cirrhosis tissues.

Results: γ-OHPdG levels in the cancerous tissues were significantly higher compared to adjacent tissues (P < 0.001) and also higher than the ones from the tissues of cirrhosis patients. Along with tumor size, histological grade, MVI grade, T stage, the percentage of ki67-positive cells and HCC progression, γ-OHPdG levels in cancerous tissues showed a gradually increasing trend. Moreover, prognostic analysis showed that higher γ-OHPdG levels in cancerous tissues were strongly correlated with lower overall survival (P < 0.001), lower intrahepatic recurrence-free survival (P < 0.001) and lower distant metastasis-free survival (P < 0.05). There was a trend, although not statistically significant, of increased levels of γ-OHPdG in cirrhosis cases that advanced to HCC, whereas γ-OHPdG levels reversely correlated with the period of time observed for cirrhosis advanced to HCC.

Conclusions: These results suggest that γ-OHPdG is a prognostic biomarker for predicting outcomes in HCC, and may serve as a prospective indicator for predicting HCC in cirrhosis patients.

## INTRODUCTION

Hepatocellular carcinoma (HCC), a major histologic liver cancer, is one of the worldwide leading causes of cancer related mortalities [1, 2]. HCC is predominant in Asia, with China accounting for nearly half of the world’s HCC cases [3]. HCC generally progresses rapidly with high invasiveness, and its 5-year relative survival rate is a mere 18% [4]. Moreover, metastasis and recurrence are attribute over 90% of HCC deaths [5]. Thus, it is critical to identify specific and reliable biomarkers to detect early-stage HCC and reduce HCC-related mortality.

HCCs develop mainly in cirrhotic livers, and chronic inflammation is the major underlying hepatocarcinogenesis cause [6–8]. In China, the prominent etiology for chronic liver inflammation is viral hepatitis (especially HBV and HCV) [9, 10], with more than 85% of HCC being predominantly related to HBV infection [11]. It has been well-established that chronic inflammation leads to oxidative stress and lipid peroxidation (LPO), producing highly reactive α, β-unsaturated aldehydes (enals) and consequently forming promutagenic cyclic DNA adducts, a significant stage in early oncogenesis [12–14].

γ-hydroxy-1,N^2^-propanodeoxyguanosine (γ-OHPdG) is ubiquitously detected as a source of endogenous DNA damage, and is one of the most abundant LPO-derived DNA adducts in mammalian tissues [15–17]. γ-OHPdG is mutagenic, known to induce predominantly DNA G to T and G to A base mutations [18, 19]. Previous studies have showed that γ-OHPdG formation principally occurs at *TP53,* a tumor suppressor gene in human cancers [20, 21], more specifically at the mutation hotspots identified in HCC, comprising a location of known HCC-specific mutations [22, 23]. Thus, it is likely that γ-OHPdG’s role in hepatocarcinogenesis may be crucial.

There have been limited reports of γ-OHPdG’s association with HCC development and progress in tissues from patients to date. Using an animal model, Fu et al. [24] investigated γ-OHPdG as a potential hepatocarcinogenesis biomarker and also as an antioxidant biomarker for cancer prevention [15]. They demonstrated that liver γ-OHPdG levels consistently correlated with HCC occurrence and progression, and anti-oxidation treatment suppressed liver tissue γ-OHPdG levels and prevented liver from carcinogenesis in a nucleotide excision repair (NER)-deficient mouse model. Furthermore, based on liver samples from HCC patients, γ-OHPdG can be used as a highly reliable predictive indicator of HCC recurrence and survival. Coia et al. also examined the formation of γ-OHPdG across all stages in HCC development in order to understand its potential role, and showed that γ-OHPdG might be a mutagenic DNA damage source in the HCC progression [25]. However, it should be noted that the cohort samples in both studies are very small. In addition, results from one previous study were not consistent with above mentioned results, which indicated high γ-OHPdG levels in paraneoplastic noncancerous tissues but not in cancerous tissues, and were highly correlated with lower distant metastasis-free survival in HCC patients [26]. Thus, the potential clinical application of γ-OHPdG in relationship to HCC progression remains inconclusive and warrants further investigation. We examined the γ-OHPdG levels in 228 HCC tissues and 46 cirrhosis tissues with the majority of the patients suffering from HBV infection in the past.

## MATERIALS AND METHODS

### Patient samples

Formalin-fixed paraffin embedded (FFPE) samples of primary HCC were acquired from 228 patients undergoing curative surgery at The First Affiliated Hospital (from January 2010 to December 2016) and The Affiliated Provincial Hospital of Shandong First Medical University (from July 2010 to August 2016). The inclusion criteria include the following aspects: complete clinicopathological characteristics, histologically confirmed HCC, no preoperative anti-tumor therapy, no other malignant tumors or fatal comorbidities and regular follow-up. Histological observations of all specimens were reassessed by experienced pathologists in accordance with the “Evidence-based Practice Guidelines for Standardized Pathological Diagnosis of Primary Liver Cancer in China: 2015 Update” [27]. Out of the 228 primary HCC individuals, 46 FFPE samples with recurrence and 28 samples with metastasis were obtained either during subsequent surgical resection or biopsy. In addition, FFPE cirrhosis specimens were obtained from 46 patients who received splenectomy and wedge-shaped liver biopsy at The First Affiliated Hospital of Shandong First Medical University from March 2005 to March 2016.

### Immunohistochemistry (IHC) staining

FFPE serial sections of the liver tissues were stained with an anti-γ-OHPdG monoclonal antibody (a kind gift from Dr Fung-Lung Chung, Department of Oncology, Lombardi Comprehensive Cancer Center, Georgetown University Medical Center, Washington DC, USA) according to previously reported protocols [25, 26]. Briefly, the slides were incubated with 3% hydrogen peroxide and 10% goat serum in phosphate buffered saline (PBS) for 10 minutes. Subsequently, the tissue sections were incubated with the γ-OHPdG antibody (1:500) at room temperature (RT) for 1 hour. Finally, the slides were treated with 3% hydrogen peroxide once again and incubated with anti-mouse horseradish peroxidase-labeled polymer for 30 minutes at RT, chromogenic reaction with DAB. Positive γ-OHPdG signals show brown-colored nuclei.

The results of IHC were semi-quantified using the histoscore and was performed by 2 independent pathologists who were blinded to all other study related data. The H-score for γ-OHPdG level on all sections of the cancerous tissues, adjacent tissues and cirrhosis tissues was graded by adding the intensity and the proportion of brown nuclear stained cells as described previously [25, 28]. Adjacent tissues were defined as non-cancerous tissues adjacent to the cancerous tissue in the same slide, which were normal, fibrosis or cirrhosis (most of them were cirrhosis). The intensity of γ-OHPdG was graded as negative (0), weak (1+), moderate (2+) or intense (3+). The distribution of positive cells was recorded in percentages, respectively. H-score was obtained by multiplying the intensity grades by the percentage of positive cells. These H-scores have a range of possible scores between 0 and 300. The median H-score of all specimens was used as the cut-off value and all specimens were divided into low and high two groups for further correlation analysis.

### Laboratory tests and follow-up

Participants were followed up with via outpatient visits and telephone calls. Pre- and postsurgical follow-up assessments including laboratory measurements such as levels of serum alpha-fetoprotein (AFP), abdominal ultrasonography and computed tomography (CT) or magnetic resonance imaging (MRI) were performed as complementary examination. Patients were evaluated once every two months during the first two years post-discharge, then once every three to six months thereafter. The follow-up period was terminated in March 2022, and cirrhosis patients were followed after diagnosis for at least 8 years.

Recurrence/metastasis was identified through imaging examinations and AFP level tests, and others were confirmed using re-surgical excision or biopsy. The study endpoints were intrahepatic recurrence-free survival (RFS), distant metastasis-free survival (MFS), and overall survival (OS). The OS was measured from the date of pathological diagnosis to the date of death or the last follow-up. RFS and MFS were recorded from the date of pathological diagnosis to recurrence and metastasis, respectively, or to the date of last follow-up.

### Statistical analysis

Analyses were performed using SPSS software program (version 17.0) and GraphPad Prism V.9 (GraphPad Prism Software, USA). Categorical variables were expressed as numbers, percentages and were performed by the chi-squared test or Fisher’s exact test. Continuous variables were reported as the mean ± standard deviation, and were compared using the *t* test or Mann-Whitney *U*-test. Wilcoxon rank-sum test was used for two-group comparisons and the Kruskal-Wallis test was applied for normalizing multiple groups. Kaplan-Meier survival curves and log-rank tests, together with the time-related receiver operating feature curve (ROC) analysis, were conducted to evaluate survival outcomes and to assess the predictive ability. Cox’s proportional hazard model was employed to univariate and multivariate analysis to obtain any independent risk factors that were related to survival. P value less than 0.05 was considered statistically significant.

## RESULTS

### Patient characteristics and the expression of γ-OHPdG

**Figure 1:**
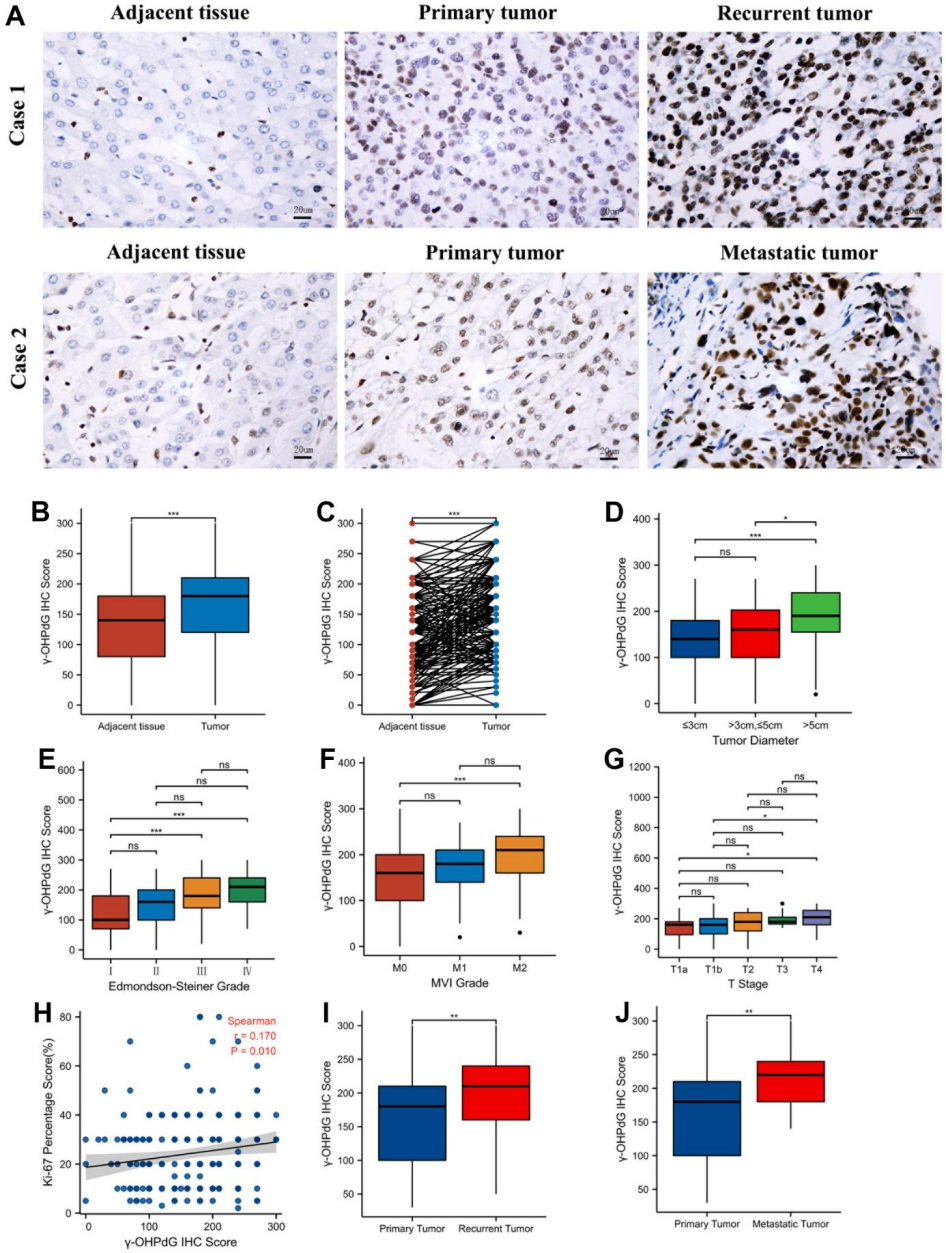
**The levels of γ-OHPdG by IHC staining in the development of HCC.** (**A**) The levels of γ-OHPdG in the adjacent tissues, primary tumors, the recurrent and metastatic tumor tissues (20×); (**B**, **C**) Comparison of the levels of γ-OHPdG in HCC and the adjacent tissues; (**D**–**G**) The histogram plot shows γ-OHPdG levels in tumor size, Edmondson-Steiner grades I–IV, MVI grades 0-2, T stages T1a-T4 HCC patients, respectively; (**H**) Dot plot between γ-OHPdG levels and ki67 percentage scores; (**I**, **J**) γ-OHPdG levels between the recurrent tumors/metastatic tumors and their primary HCC tissues.*P < 0.05, **P < 0.01, ***P < 0.001 (Wilcoxon rank sum test and Kruskal-Wallis test).

The baseline characteristics for the HCC patients are summarized in Table 1. The 228 FFPE liver samples were evaluated for γ-OHPdG staining using IHC, and all cancerous and the adjacent tissues were scored by histological evaluation as described above. The presence of γ-OHPdG was detected in the nuclei in the adjacent tissues and carcinomatous tissues as shown in Figure 1A. The levels of γ-OHPdG were significantly increased in carcinomatous tissues compared with those in the adjacent tissues (P < 0.001) (Figure 1B). Wilcoxon signed rank test showed similar results (Figure 1C). Of the 228 resected specimens, 85 (37%) were grouped with lower and 143 (63%) with higher levels of γ-OHPdG, and were analyzed accordingly. Our data showed that the γ-OHPdG levels were associated with the tumor size, histological grades (ES), MVI grades and T stages (Figure 1D–1G). γ-OHPdG levels in HCC were also positively correlated with the percentage of ki67-positive cells (Figure 1H, p = 0.010). Comparison of the patient’s clinicopathological characteristics with the two groups, significant correlation was found between γ-OHPdG levels and presurgical AFP levels (Table 1), but other baseline variables including age, sex, HbsAg, HBV DNA load, ALT, AST, TBIL, prothrombin time, alcoholism, long-term smoking, HCC family history, surgical procedures, nodule numbers, tumor capsule, surgical margin, neoplastic necrosis, satellite nodules and background liver function, were not associated. No significant difference in macrovascular invasion between the two groups was observed.

**Table 1:** Baseline clinicopathological characteristics of hepatocellular carcinoma patients based on γ-OHPdG levels.

**Characteristics**	**γ-OHPdG levels**		**P-value**
	**Low(n=85)**	**High(n=143)**	
Age, mean ± SD	55.73 ± 8.58	53.21 ± 9.9	0.052
Sex, n (%)			0.483
Female	15 (6.6%)	19 (8.3%)	
Male	70 (30.7%)	124 (54.4%)	
HbsAg, n (%)			0.091
Negative	18 (7.9%)	17 (7.5%)	
Positive	67 (29.4%)	126 (55.3%)	
HBV DNA load, n (%)			0.180
≤5×10^2^ IU/ml	56 (24.6%)	80 (35.1%)	
>5×10^2^ IU/ml	29 (12.7%)	63 (27.6%)	
AFP, ng/ml, n (%)			**0.024**
≤20	36 (15.8%)	42 (18.4%)	
20	400	31 (13.6%)	47 (20.6%)
>400	18 (7.9%)	54 (23.7%)	
ALT levels, n (%)			0.381
Normal	58 (25.4%)	88 (38.6%)	
High	27 (11.8%)	55 (24.1%)	
AST levels, n (%)			1.000
Normal	15 (6.6%)	26 (11.4%)	
High	70 (30.7%)	117 (51.3%)	
TBIL levels, n (%)			0.523
Normal	62 (27.2%)	111 (48.7%)	
High	23 (10.1%)	32 (14%)	
PT levels, n (%)			0.872
Normal	50 (21.9%)	87 (38.2%)	
High	35 (15.4%)	56 (24.6%)	
Alcoholism, n (%)			1.000
No	63 (27.6%)	107 (46.9%)	
Yes	22 (9.6%)	36 (15.8%)	
Long term smoking, n (%)			0.926
No	56 (24.6%)	92 (40.4%)	
Yes	29 (12.7%)	51 (22.4%)	
HCC family history, n (%)			0.877
No	64 (28.1%)	105 (46.1%)	
Yes	21 (9.2%)	38 (16.7%)	
Liver transplantation, n (%)			1.000
No	77 (33.8%)	130 (57%)	
Yes	8 (3.5%)	13 (5.7%)	
Tumor number, n (%)			0.524
1 nodule	72 (31.6%)	115 (50.4%)	
≥2 nodules	13 (5.7%)	28 (12.3%)	
Tumor diameter, n (%)			**< 0.001**
<5cm	57 (25%)	59 (25.9%)	
≥5cm	28 (12.3%)	84 (36.8%)	
Tumor capsule, n (%)			0.178
Present	76 (33.3%)	117 (51.3%)	
Absent	9 (3.9%)	26 (11.4%)	
Surgical margin, n (%)			0.219
Negative	74 (32.5%)	114 (50%)	
Positive	11 (4.8%)	29 (12.7%)	
Neoplastic necrosis, n (%)			0.306
No	65 (28.5%)	99 (43.4%)	
Yes	20 (8.8%)	44 (19.3%)	
ES grade, n (%)			**0.004**
I-II	56 (24.6%)	65 (28.5%)	
III-IV	29 (12.7%)	78 (34.2%)	
MVI grade, n (%)			**0.003**
M0	61 (26.8%)	70 (30.7%)	
M1	13 (5.7%)	34 (14.9%)	
M2	11 (4.8%)	39 (17.1%)	
Macrovascular invasion, n (%)			0.064
Negative	81 (35.5%)	124 (54.4%)	
Positive	4 (1.8%)	19 (8.3%)	
Satellite nodules, n (%)			0.915
Absent	79 (34.6%)	131 (57.5%)	
Present	6 (2.6%)	12 (5.3%)	
Liver cirrhosis, n (%)			1.000
No	11 (4.8%)	19 (8.3%)	
Yes	74 (32.5%)	124 (54.4%)	
Ki67, median (IQR)	0.2 (0.1, 0.3)	0.2 (0.1, 0.4)	**0.033**

Statistically significant p values are in bold (p < 0.05).

Note: Long-term smoking was defined as smoking at least 10 cigarettes a day for more than 10 years.

### The co-relationship between the levels of γ-OHPdG and HCC development

In addition to the 228 FFPE liver samples, 46 recurrent HCC and 23 metastatic HCC samples were also examined by IHC staining and scored by histological assessment as described in Materials and Methods, and the results are shown in Figure 1A. The γ-OHPdG levels in the recurrent or metastatic tumors were compared to the levels in the corresponding primary HCC. It was observed that γ-OHPdG levels were significantly higher in the recurrent or metastatic tumors, respectively (Figure 1I, 1J, P < 0.01 for both).

### γ-OHPdG as a useful prognostic biomarker for HCC patients

The median follow-up time of HCC patients was 52.5 months for the present study. The average time to postoperative recurrence was 26.8 months, the average time to postoperative metastasis was 23.6 months, and the average OS was 53.6 months.

**Figure 2:**
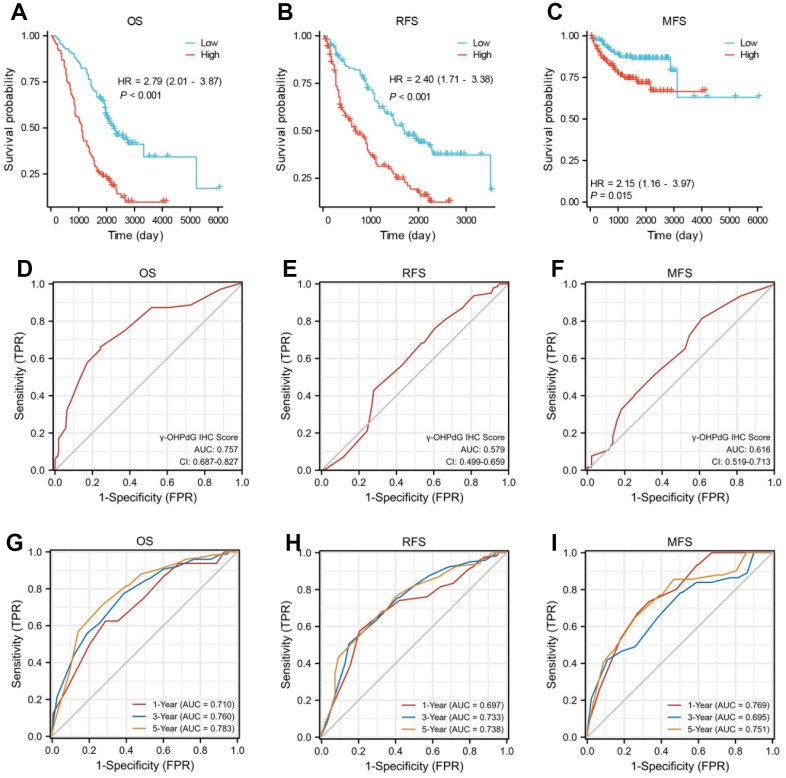
**The γ-OHPdG levels and the prediction of prognosis in HCC patients.** (**A**–**C**) Kaplan-Meier survival curve analysis of OS, PFS and MFS rates with high and low γ-OHPdG levels in HCC patients, respectively; (**D**–**F**) ROC curve validation of the prognostic value of the γ-OHPdG; (**G**–**I**) Time-dependent ROC curve analysis of the γ-OHPdG levels for OS, MFS and RFS.

As shown in Figure 2A–2C and Supplementary Tables 1–3, higher γ-OHPdG levels (median value as the cut-off) in cancerous tissues were correlated reversely with overall survival (OS, log-rank P < 0.001), intrahepatic recurrence-free survival (RFS, log-rank P < 0.001), and metastasis-free survival (MFS, log-rank P = 0.015). Kaplan-Meier plot with log-rank analyses also indicated that higher levels of γ-OHPdG in the adjacent tissues, similar to cancerous tissues, was associated with shorter OS (P < 0.001), shorter RFS (P < 0.001) and shorter MFS (P < 0.001) as shown in Supplementary Figure 1A–1C. In addition, receiver operating characteristic (ROC) curve and area under the curve (AUC) statistical analysis were performed to evaluate the capacity of γ-OHPdG levels in carcinomatous tissues and the adjacent tissues for predicting the OS, MFS and RFS of HCC. The AUC of the ROC curve was 0.757, 0.616 and 0.579, respectively (Figure 2D–2F). Figure 2G–2I show the predictive potential of the γ-OHPdG in carcinomatous tissues using time-dependent ROC curves. And the area under the ROC curve (AUC) of the prognostic model for OS are 0.710 at 1 year, 0.760 at 3 years and 0.783 at 5 years, and an AUC of 0.769 at 1 year, 0.695 at 3 years and 0.751 at 5 years for MFS, respectively. Furthermore, the AUC of the prognostic model for RFS at 1, 3 and 5 years are 0.697, 0.733 and 0.738, respectively. Supplementary Figure 2D–2F shows the predictive potential of the γ-OHPdG in the adjacent tissues using time-dependent ROC curves. The AUC of the prognostic model for OS are 0.727 at 1 year, 0.785 at 3 years and 0.802 at 5 years, and an AUC of 0.773 at 1 year, 0.763 at 3 years and 0.759 at 5 years for MFS, respectively. Furthermore, the AUC of the prognostic model for RFS at 1, 3 and 5 years are 0.728, 0.669 and 0.727, respectively.

Based on these observation, Cox proportional hazard regression analysis were implemented to identify independent predictors of OS, RFS and MFS, and the results are shown in Supplementary Tables 1–3. The prognostic factors for OS based on multivariable analyses were multinodular tumor (HR 2.045, 95% CI 1.363-3.069, P < 0.001), larger tumor diameter (HR 2.104, 95% CI 1.415-3.127, P < 0.001), grade MVI-M1 (HR 2.023, 95% CI 1.281-3.194, P = 0.003), grade MVI-M2 (HR 2.070, 95% CI 1.189-3.602, P = 0.010), MVI-TTG classification (M1,HR 2.023, 95% CI 1.281-3.194, P = 0.003;M2,HR 2.070, 95% CI 1.189-3.602, P = 0.010), positive of TBIL (HR 1.485, 95% CI 1.005-2.194, P = 0.047) and γ-OHPdG higher levels (HR 1.011, 95% CI 1.008-1.014, P < 0.001). Whereas the multivariable Cox-regression analyses of MFS indicated that the prognostic factors were liver transplantation (HR 2.337, 95% CI 1.004-5.443, P = 0.049), positive of AST (HR 5.993, 95% CI 1.352-26.561, P = 0.018), larger tumor diameter (HR 2.645, 95% CI 1.284-5.446, P = 0.008), grade MVI-M1(HR 2.763, 95% CI 1.281-5.963, P = 0.010), macrovascular invasion (HR 2.798, 95% CI 1.008-7.766, P = 0.048), higher Ki67 percentage (HR20.337, 95% CI 1.590-260.160, P = 0.021) and γ-OHPdG higher levels (HR 1.008, 95% CI 1.003-1.014, P = 0.002). Furthermore, only γ-OHPdG level in cancerous tissue served as an independent prognostic factor associated with intrahepatic recurrence-free survival (HR 1.007, 95% CI 1.004-1.009, P < 0.001).

### The expression levels of γ-OHPdG in cirrhosis patients and its relationship with the time of cirrhosis advanced to HCC

**Figure 3:**
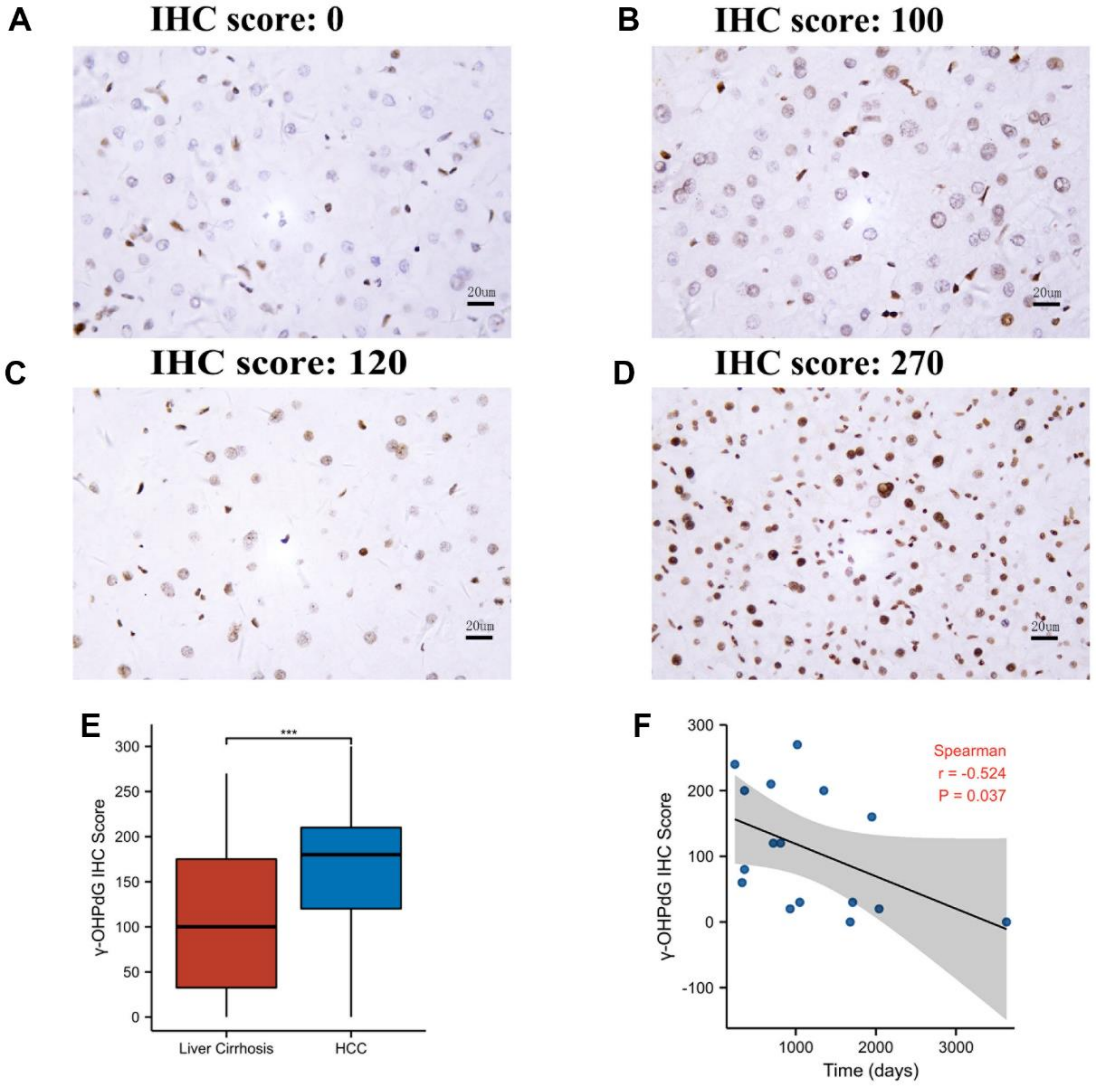
**The γ-OHPdG levels in cirrhosis patients.** (**A**, **B**) The levels of γ-OHPdG (representatives of IHC staining) in cirrhosis tissues. (**C**, **D**) γ-OHPdG IHC examination of cirrhosis tissues advanced to HCC; (**E**) The comparison of γ-OHPdG levels in HCC and the cirrhosis tissues; (**F**) Dot plotting and Spearman fitting show the correlation between γ-OHPdG levels and the time of cirrhosis advanced to HCC. *** indicates P < 0.001 (Wilcoxon rank sum test).

Out of the 46 cirrhosis individuals, 16 patients progressed to HCC during follow-up, and the median time advanced to HCC time was 32.5 months. The presence of γ-OHPdG in cirrhosis tissues was detected with IHC as shown in Figure 3A–3D. The levels of γ-OHPdG were generally higher in cirrhosis tissues advanced to HCC (Figure 3C, 3D) except in 2 out of 16 samples where γ-OHPdG levels were extremely low, thus resulting in no significant difference between these two groups (Supplementary Figure 2). We also compared γ-OHPdG levels between these 46 cirrhosis patients and the above mentioned 228 HCC patients. The levels of γ-OHPdG in HCC tissues were significantly higher than that in cirrhosis tissues (P < 0.001) (Figure 3E). Further analysis showed that γ-OHPDG levels were reversely correlated with the time course of cirrhosis advanced to HCC (P = 0.037) (Figure 3F).

## DISCUSSION

There has yet to be a significant clinical improvement in the discovery of biomarkers for HCC surveillance and early diagnosis in the last few decades [29]. Thus, HCC is often diagnosed at advanced stages, leading to not only a poor prognosis, but also high mortality. Thus, exploring and identifying new biomarkers that could detect HCC earlier and predict its prognosis accurately is essential and critical.

Several biomarkers have already been proposed [30–35], but their clinical utility has not been widely accepted. It is well known that HCC is an inflammation-related cancer, and HCC risks are associated with chronic inflammation and its resultant oxidative stress [36]. Moreover, it has been proved that oxidative stress is involved in HCC migration, invasion and metastasis [37]. These findings imply that biomarkers associated with oxidative stress may serve as potential HCC prognostic indicators. Unfortunately, only a few studies on the correlation between oxidative stress markers and HCC have been reported and the findings so far remain inconclusive.

γ-OHPdG, a pro mutagenic DNA adduct derived from acrolein as a product of oxidative stress and LPO, has been involved in cancer development [19, 20]. Therefore, the present study investigated the association between γ-OHPdG levels and the HCC clinical characteristics including prognosis of HCC in Chinese patients. Our results showed that the levels of γ-OHPdG were higher in HCC cancerous tissues comparing to the ones in adjacent tissues (majority of them were cirrhosis), which is consistent with the previous study conducted by Feng et al. [26], but not with the study by Fu et al. [24] where they did not observe significant difference of γ-OHPdG levels between the two tissues. Differences in etiology might contribute to the inconsistency. HBV is the leading cause of HCC in China, and about 94% of HCC cases in this study are predominantly related to HBV infection, whereas in North America HCV, unhealthy alcohol use, and non-alcoholic steatohepatitis (NASH) are the main causes of HCC [38]. The population of study subject by Fu et al. Previous findings demonstrated that NER is the main DNA repair system for repairing cyclic 1, N^2^-propanodeoxyguanine DNA adducts-induced DNA damage [39]. Further studies demonstrate that the inhibition of xeroderma pigmentosum type B (XPB) and type D (XPD) helicase in patients with HBV resulted in the hepatitis B virus X protein (HBx) suppressing the NER pathway, leading to inefficient removal of DNA adducts [40]. As Feng et al. [26] speculated, HBV infection causes both DNA mutation and repair pathway disruption, thus resulting in an additive effect on HCC progression. In addition, we found that γ-OHPdG levels were significantly higher in the HCC tissues compared with that in the cirrhosis tissues, which is consistent with previous study by Coia et al. [25]. In agreement with the studies mentioned above, our results demonstrate that γ-OHPdG levels are indicative for oxidative stress, and may serve as a predictor of DNA damage-mediated hepatocarcinogenesis.

Since oxidative stress contributes to the progression of liver disease, we expected an increase in the DNA damage and oxidative stress with the increase of HCC stage, recurrence and metastasis [41]. Fu et al. [15] demonstrated that the relationship between γ-OHPdG levels in liver DNA and HCC development, and significantly decreased γ-OHPdG levels are associated with antioxidant treatment and notably decreased HCC incidence in animal models. Our present study provides evidence that γ-OHPdG levels in cancerous tissues increase as HCC progresses in regards to tumor size, grade, stage and tumor cell proliferation status as indicated by Ki67 staining, and is significantly higher in recurrent or metastatic tumors compared to the levels in the corresponding primary HCC as well. These findings suggest that γ-OHPdG may serve as a reliable indicator of the DNA damage levels for the prediction of HCC progression (recurrence and metastasis).

The above findings were also consistent with the results of survival analysis and logistic regression, which indicate higher levels of γ-OHPdG both in the carcinomatous tissues and the adjacent tissues of HCC patients are associated with shorter overall survival, shorter intrahepatic recurrence-free survival and shorter distant metastasis-free survival, suggesting that oxidative stress may be involved in HCC migration, invasion, and metastasis. Presumably, oxidative stress induced γ-OHPdG accumulation could trigger cancerous cell transformation and proliferation, resulting in distant metastasis, recurrence, and shorter overall survival. More importantly, the results of the multivariate Cox analysis demonstrated that γ-OHPdG is an independent prognostic biomarker for HCC, and the ROC curve suggests the potential value of γ-OHPdG levels in predicting HCC prognosis.

Cirrhosis is a major HCC risk factor and up to 15% of cirrhosis cases each year advanced to HCC [42, 43]. The HCC autopsy results indicated that 80–90% had underlying cirrhosis [44]. Thus, it is clinically valuable to classify and monitor the risk of liver cirrhosis in order to prevent hepatocarcinogenesis. As previous studies indicated, γ-OHPdG may lead to hepatocarcinogenesis. To evaluate the association between γ-OHPdG levels with different stages of cirrhosis, a set of 46 samples were examined. During the period of follow-up, 16 of the 46 patients advanced to HCC. Out of these 16 patients, 14 patients had higher levels of γ-OHPdG in their cirrhosis tissues compared to those without advanced to HCC. We re-sliced and stained the remaining 2 cirrhosis specimens with low γ-OHPdG levels to identify potential technical errors, tissue degradation, or specific clincopathological characteristic differences, but concluded that there was no reasonable explanation for their extremely low γ-OHPdG levels.

There are certain limitations in this study. Firstly, the immunohistochemistry staining technique used in this study is a semi-quantitative method that may be subject to observer bias. Secondly, the sample size of cirrhosis patients in our study was small and the subject follow-up periods are not long enough.

## CONCLUSIONS

Our current study evaluates the prognostic value and clinical characteristics of γ-OHPdG levels in HCC and cirrhosis patients by evaluating the clinical characteristics of γ-OHPdG levels in HCC patients. The findings demonstrate that γ-OHPdG levels were upregulated in HCC specimens and positively correlated with the progression of HCC. Further analysis showed that γ-OHPdG is an independent negative predictor for OS, MFS and RFS in HCC patients. However, due to the small sample size, it should be noted that the clinical use of γ-OHPdG as an HCC diagnostic and prognostic biomarker needs further investigation with a larger prospective cohort study.

## Supplementary Material

Supplementary Figures

Supplementary Tables
