# Supplementary material for: The lipid peroxidation-derived DNA adduct γ-OHPdG as a diagnostic and prognostic biomarker in hepatocellular carcinoma
Source: Aging (Albany NY). 2023 Jul 28;15(14):7258–77. doi: 10.18632/aging.204910 (PMC10415556; doi:10.18632/aging.204910)
Supplement: Supplementary Figures [file aging-15-204910-s001.pdf]

SUPPLEMENTARY FIGURES

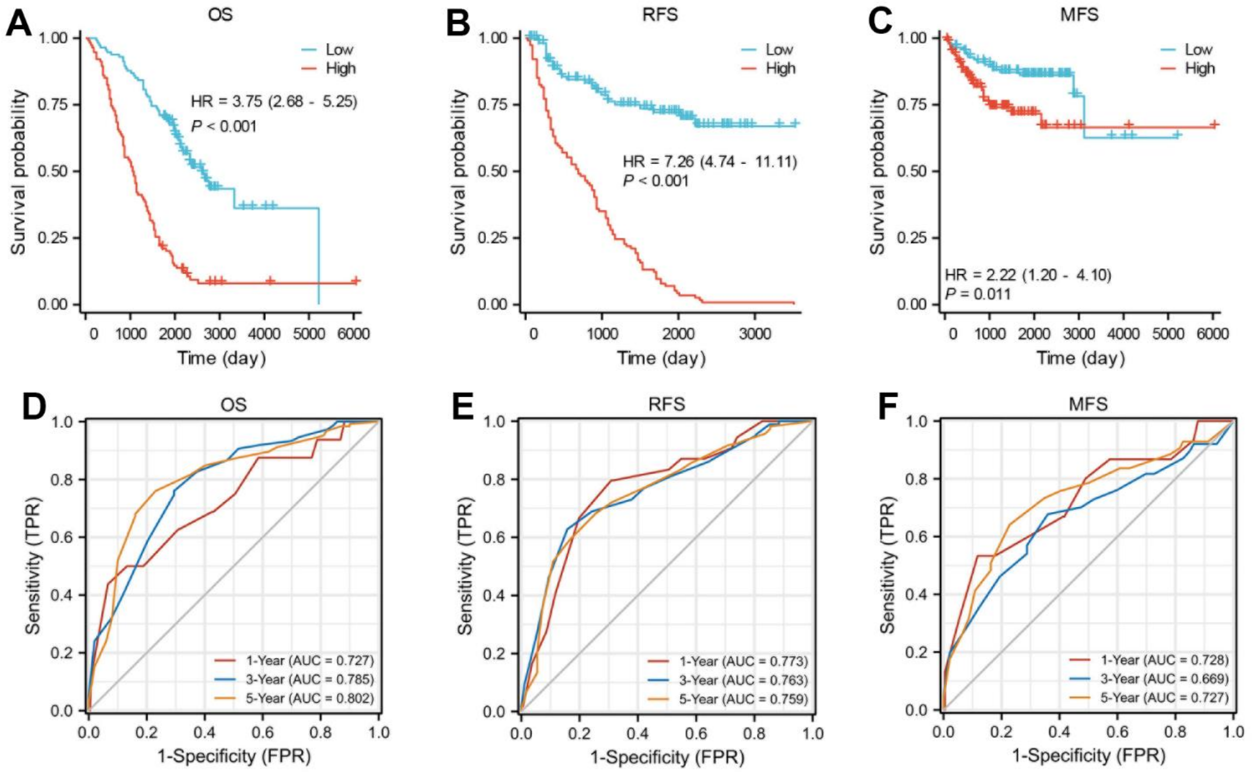

Supplementary Figure 1. The γ-OHPdG levels and prediction of prognosis in adjacent tissues. (**A**–**C**) Kaplan-Meier survival curve analysis shows OS, PFS and MFS rates of high and low γ-OHPdG levels in adjacent tissues, respectively. (**D**–**F**) Time-dependent ROC curve analysis of the γ-OHPdG levels for OS, MFS and RFS.

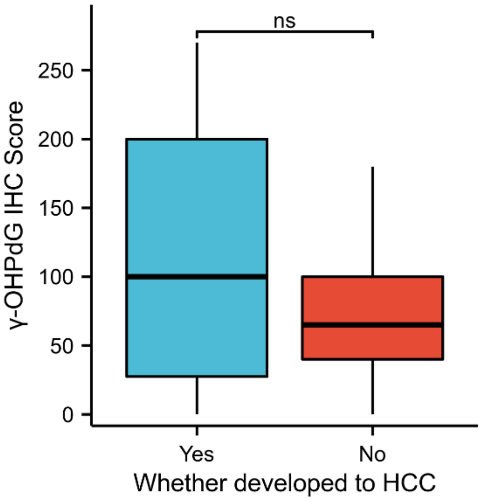

Supplementary Figure 2. Comparison of the levels of γ-OHPdG in the cirrhosis tissues advanced to HCC and not advanced to HCC.
