# Supplementary material for: The lipid peroxidation-derived DNA adduct γ-OHPdG as a diagnostic and prognostic biomarker in hepatocellular carcinoma
Source: Aging (Albany NY). 2023 Jul 28;15(14):7258–77. doi: 10.18632/aging.204910 (PMC10415556; doi:10.18632/aging.204910)
Supplement: Supplementary Tables [file aging-15-204910-s002.pdf]

SUPPLEMENTARY TABLES

Supplementary Table 1. Univariate and multivariate analysis of overall survival for hepatocellular carcinoma.

Characteristics	Total(N)	Univariate analysis		Multivariate analysis	
		Hazard ratio (95% CI)	P value	Hazard ratio (95% CI)	P value
Age	228	1.001 (0.984-1.018)	0.903		
Sex	228				
Female	34	Reference			
Male	194	1.282 (0.808-2.035)	0.291		
HbsAg	228				
Negative	35	Reference			
Positive	193	1.207 (0.775-1.880)	0.405		
HBV DNA load	228				
≤5×10^2 IU/ml	136	Reference			
>5×10^2 IU/ml	92	1.328 (0.967-1.823)	0.079	0.865 (0.603-1.240)	0.430
AFP, ng/ml	228				
≤20	78	Reference			
20-400	78	1.217 (0.822-1.802)	0.327	0.907 (0.600-1.372)	0.644
>400	72	1.741 (1.186-2.555)	**0.005**	0.880 (0.564-1.373)	0.574
ALT levels	228				
Normal	146	Reference			
High	82	1.285 (0.931-1.772)	0.127		
AST levels	228				
Normal	41	Reference			
High	187	1.581 (1.015-2.464)	**0.043**	1.341 (0.844-2.130)	0.214
TBIL levels	228				
Normal	173	Reference			
High	55	1.402 (0.986-1.995)	0.060	1.485 (1.005-2.194)	**0.047**
PT levels	228				
Normal	137	Reference			
High	91	1.356 (0.985-1.866)	0.061	1.196 (0.832-1.718)	0.334
Alcoholism	228				
No	170	Reference			
Yes	58	1.046 (0.732-1.494)	0.805		
Long-term smoking	228				
No	148	Reference			
Yes	80	1.243 (0.898-1.719)	0.190		
HCC family history	228				
No	169	Reference			
Yes	59	1.030 (0.719-1.476)	0.870		
Liver transplantation	228				
No	207	Reference			
Yes	21	1.463 (0.883-2.422)	0.140		
Tumor number	228				
1 nodule	187	Reference			
≥2 nodules	41	1.665 (1.137-2.437)	**0.009**	2.045 (1.363-3.069)	**<0.001**
Tumor diameter	228				
<5cm	116	Reference			
≥5cm	112	2.854 (2.059-3.954)	**<0.001**	2.104 (1.415-3.127)	**<0.001**

Tumor capsule	228				
Present	193	Reference			
Absent	35	1.558 (1.051-2.310)	**0.027**	1.490 (0.964-2.303)	0.073
Surgical margin	228				
Negative	188	Reference			
Positive	40	1.187 (0.787-1.790)	0.413		
Neoplastic necrosis	228				
No	164	Reference			
Yes	64	1.825 (1.307-2.549)	**<0.001**	1.124 (0.763-1.658)	0.554
E-S grade	228				
I-II	121	Reference			
III-IV	107	2.778 (2.013-3.835)	**<0.001**	1.239 (0.774-1.982)	0.372
MVI grade	228				
M0	131	Reference			
M1	47	2.830 (1.903-4.208)	**<0.001**	2.023 (1.281-3.194)	**0.003**
M2	50	4.152 (2.837-6.076)	**<0.001**	2.070 (1.189-3.602)	**0.010**
Macrovascular invasion	228				
Negative	205	Reference			
Positive	23	2.719 (1.721-4.296)	**<0.001**	0.842 (0.462-1.535)	0.575
Satellite nodules	228				
Absent	210	Reference			
Present	18	2.831 (1.680-4.770)	**<0.001**	1.566 (0.876-2.800)	0.130
Liver Cirrhosis	228				
No	30	Reference			
Yes	198	0.943 (0.600-1.480)	0.798		
Ki67	228	11.245 (4.329-29.208)	**<0.001**	1.666 (0.495-5.607)	0.409
γ-OHPdG IHC Score	228	1.011 (1.008-1.014)	**<0.001**	1.011 (1.008-1.014)	**<0.001**

Statistically significant p values are in bold ($p < 0.05$).

Note: Long-term smoking was defined as smoking at least 10 cigarettes a day for more than 10 years.

Supplementary Table 2. Univariate and multivariate analysis of intrahepatic recurrence-free survival for hepatocellular carcinoma.

Characteristics	Total(N)	Univariate analysis		Multivariate analysis	
		Hazard ratio (95% CI)	P value	Hazard ratio (95% CI)	P value
Age	228	1.006 (0.987-1.024)	0.552		
Sex	228				
Female	34	Reference			
Male	194	0.972 (0.621-1.521)	0.900		
HbsAg	228				
Negative	35	Reference			
Positive	193	1.167 (0.746-1.825)	0.500		
HBV DNA load	228				
≤5×10^2 IU/ml	136	Reference			
>5×10^2 IU/ml	92	1.440 (1.032-2.009)	**0.032**	1.137 (0.787-1.644)	0.494
AFP, ng/ml	228				
≤20	78	Reference			
20-400	78	1.493 (0.993-2.244)	0.054	1.260 (0.826-1.924)	0.283
>400	72	1.916 (1.264-2.907)	**0.002**	1.239 (0.778-1.974)	0.367
ALT levels	228				
Normal	146	Reference			
High	82	1.152 (0.814-1.631)	0.425		
AST levels	228				
Normal	41	Reference			
High	187	1.280 (0.835-1.962)	0.257		
TBIL levels	228				
Normal	173	Reference			
High	55	1.229 (0.841-1.796)	0.287		
PT levels	228				
Normal	137	Reference			
High	91	1.059 (0.755-1.486)	0.738		
Alcoholism	228				
No	170	Reference			
Yes	58	1.052 (0.725-1.526)	0.790		
Long-term smoking	228				
No	148	Reference			
Yes	80	1.174 (0.833-1.656)	0.360		
HCC family history	228				
No	169	Reference			
Yes	59	0.981 (0.667-1.443)	0.922		
Liver transplantation	228				
No	207	Reference			
Yes	21	0.731 (0.384-1.391)	0.339		
Tumor number	228				
1 nodule	187	Reference			
≥2 nodules	41	1.312 (0.862-1.996)	0.205		
Tumor diameter	228				
<5cm	116	Reference			
≥5cm	112	1.706 (1.222-2.382)	**0.002**	1.197 (0.806-1.777)	0.374
Tumor capsule	228				
Present	193	Reference			

Absent	35	1.491 (0.959-2.318)	0.076	1.345 (0.849-2.130)	0.207
Surgical margin	228				
Negative	188	Reference			
Positive	40	1.264 (0.825-1.937)	0.283		
Neoplastic necrosis	228				
No	164	Reference			
Yes	64	1.297 (0.896-1.878)	0.168		
E-S grade	228				
I-II	121	Reference			
III-IV	107	1.695 (1.213-2.368)	**0.002**	1.083 (0.726-1.616)	0.696
MVI grade	228				
M0	131	Reference			
M1	47	1.774 (1.158-2.720)	**0.008**	1.406 (0.881-2.246)	0.153
M2	50	2.355 (1.547-3.585)	**<0.001**	1.388 (0.791-2.435)	0.254
Macrovascular invasion	228				
Negative	205	Reference			
Positive	23	2.508 (1.441-4.366)	**0.001**	1.386 (0.705-2.725)	0.344
Satellite nodules	228				
Absent	210	Reference			
Present	18	2.418 (1.291-4.529)	**0.006**	1.652 (0.861-3.170)	0.131
Liver Cirrhosis	228				
No	30	Reference			
Yes	198	1.094 (0.675-1.775)	0.715		
Ki67	228	1.817 (0.606-5.445)	0.287		
γ-OHPdG IHC Score	228	1.008 (1.005-1.011)	**<0.001**	1.007 (1.004-1.009)	**<0.001**

Statistically significant p values are in bold ($p < 0.05$).

Note: Long-term smoking was defined as smoking at least 10 cigarettes a day for more than 10 years.

Supplementary Table 3. Univariate and multivariate analysis of distant metastasis-free survival for hepatocellular carcinoma.

Characteristics	Total(N)	Univariate analysis		Multivariate analysis	
		**Hazard ratio (95% CI)**	**P value**	**Hazard ratio (95% CI)**	**P value**
Age	228	0.988 (0.957-1.019)	0.438		
Sex	228				
Female	34	Reference			
Male	194	2.002 (0.712-5.626)	0.188		
HbsAg	228				
Negative	35	Reference			
Positive	193	1.541 (0.607-3.912)	0.363		
HBV DNA load	228				
$\leq 5\times10^2$ IU/ml	136	Reference			
$>5\times10^2$ IU/ml	92	1.260 (0.692-2.294)	0.449		
AFP, ng/ml	228				
≤20	78	Reference			
20-400	78	0.641 (0.302-1.359)	0.246		
>400	72	1.075 (0.540-2.140)	0.836		
ALT levels	228				
Normal	146	Reference			
High	82	1.804 (0.998-3.264)	0.051	1.146 (0.573-2.292)	0.700
AST levels	228				
Normal	41	Reference			
High	187	5.357 (1.295-22.163)	**0.021**	5.993 (1.352-26.561)	**0.018**
TBIL levels	228				
Normal	173	Reference			
High	55	0.920 (0.441-1.921)	0.825		
PT levels	228				
Normal	137	Reference			
High	91	1.430 (0.790-2.592)	0.238		
Alcoholism	228				
No	170	Reference			
Yes	58	0.960 (0.485-1.900)	0.906		
Long-term smoking	228				
No	148	Reference			
Yes	80	1.062 (0.569-1.983)	0.849		
HCC family history	228				
No	169	Reference			
Yes	59	1.237 (0.647-2.365)	0.521		
Liver transplantation	228				
No	207	Reference			
Yes	21	2.060 (0.917-4.627)	0.080	2.337 (1.004-5.443)	**0.049**
Tumor number	228				
1 nodule	187	Reference			
≥2 nodules	41	0.803 (0.339-1.903)	0.619		
Tumor diameter	228				
<5cm	116	Reference			
≥5cm	112	3.425 (1.783-6.581)	**<0.001**	2.645 (1.284-5.446)	**0.008**
Tumor capsule	228				
Present	193	Reference			
Absent	35	1.769 (0.873-3.585)	0.114		

Surgical margin	228				
Negative	188	Reference			
Positive	40	1.152 (0.534-2.484)	0.719		
Neoplastic necrosis	228				
No	164	Reference			
Yes	64	1.496 (0.792-2.828)	0.215		
E-S grade	228				
I-II	121	Reference			
III-IV	107	2.124 (1.159-3.895)	**0.015**	0.702 (0.323-1.524)	0.371
MVI grade	228				
M0	131	Reference			
M1	47	3.799 (1.905-7.577)	**<0.001**	2.763 (1.281-5.963)	**0.010**
M2	50	2.599 (1.174-5.750)	**0.018**	0.581 (0.202-1.672)	0.314
Macrovascular invasion	228				
Negative	205	Reference			
Positive	23	2.483 (1.100-5.606)	**0.029**	2.798 (1.008-7.766)	**0.048**
Satellite nodules	228				
Absent	210	Reference			
Present	18	2.470 (0.967-6.308)	0.059	1.294 (0.455-3.682)	0.629
Liver Cirrhosis	228				
No	30	Reference			
Yes	198	0.796 (0.354-1.789)	0.581		
Ki67	228	14.283 (2.497-81.698)	**0.003**	20.337 (1.590-260.160)	**0.021**
γ-OHPdG IHC Score	228	1.009 (1.004-1.014)	**<0.001**	1.008 (1.003-1.014)	**0.002**

Statistically significant p values are in bold ($p < 0.05$).

Note: Long-term smoking was defined as smoking at least 10 cigarettes a day for more than 10 years.
